# RETRACTION: Hsa_circ_0005085 May Suppress Cutaneous Squamous Cell Carcinoma Growth and Metastasis Through Targeting the MiR‐186‐5p/LAMC1 Axis

**DOI:** 10.1111/srt.70181

**Published:** 2025-05-15

**RETRACTION**: L. Wang, Y. Liu, Q. Gao, and R. Hu, “Hsa_circ_0005085 May Suppress Cutaneous Squamous Cell Carcinoma Growth and Metastasis Through Targeting the MiR‐186‐5p/LAMC1 Axis,” *Skin Research and Technology* 29, no. 6 (2023): e13321, https://doi.org/10.1111/srt.13321.

The above article, published online on 13 June 2023 in Wiley Online Library (wileyonlinelibrary.com), has been retracted by agreement between *Skin Research and Technology*; and John Wiley & Sons Ltd. The article has been accepted for publication following an inadequate peer review process. Several flaws and inconsistencies between results presented and experimental methods described were found. Furthermore, duplication affecting the middle panels of Figure 2G has been identified. Accordingly, the article is retracted as the editors consider its conclusions to be invalid. The authors have been informed of the decision of retraction but unavailable for a final confirmation.

